# Association Between Oral Microbiota and Cigarette Smoking in the Chinese Population

**DOI:** 10.3389/fcimb.2021.658203

**Published:** 2021-05-28

**Authors:** Yi-Jing Jia, Ying Liao, Yong-Qiao He, Mei-Qi Zheng, Xia-Ting Tong, Wen-Qiong Xue, Jiang-Bo Zhang, Lei-Lei Yuan, Wen-Li Zhang, Wei-Hua Jia

**Affiliations:** ^1^ School of Public Health, Sun Yat‐sen University, Guangzhou, China; ^2^ State Key Laboratory of Oncology in South China, Collaborative Innovation Center for Cancer Medicine, Guangdong Key Laboratory of Nasopharyngeal Carcinoma Diagnosis and Therapy, Sun Yat‐sen University Cancer Center, Guangzhou, China

**Keywords:** oral microbiota, cigarette smoking, 16S rRNA gene sequencing, China, saliva

## Abstract

The oral microbiota has been observed to be influenced by cigarette smoking and linked to several human diseases. However, research on the effect of cigarette smoking on the oral microbiota has not been systematically conducted in the Chinese population. We profiled the oral microbiota of 316 healthy subjects in the Chinese population by 16S rRNA gene sequencing. The alpha diversity of oral microbiota was different between never smokers and smokers (*P* = 0.002). Several bacterial taxa were first reported to be associated with cigarette smoking by LEfSe analysis, including *Moryella* (*q* = 1.56E-04), *Bulleidia* (*q* = 1.65E-06), and *Moraxella* (*q* = 3.52E-02) at the genus level and *Rothia dentocariosa* (*q* = 1.55E-02), *Prevotella melaninogenica* (*q* = 8.48E-08), *Prevotella pallens* (*q* = 4.13E-03), *Bulleidia moorei* (*q* = 1.79E-06), *Rothia aeria* (*q* = 3.83E-06), *Actinobacillus parahaemolyticus* (*q* = 2.28E-04), and *Haemophilus parainfluenzae* (*q* = 4.82E-02) at the species level. Two nitrite-producing bacteria that can increase the acidity of the oral cavity, *Actinomyces* and *Veillonella*, were also enriched in smokers with FDR-adjusted *q*-values of 3.62E-06 and 1.10E-06, respectively. Notably, we observed that two acid production-related pathways, amino acid-related enzymes (*q* = 6.19E-05) and amino sugar and nucleotide sugar metabolism (*q* = 2.63E-06), were increased in smokers by PICRUSt analysis. Finally, the co-occurrence analysis demonstrated that smoker-enriched bacteria were significantly positively associated with each other and were negatively correlated with the bacteria decreased in smokers. Our results suggested that cigarette smoking may affect oral health by creating a different environment by altering bacterial abundance, connections among oral microbiota, and the microbiota and their metabolic function.

## Introduction

The human oral cavity is colonized by more than 600 different bacterial species together with viruses and fungi, which collectively compose the oral microbiota (Dewhirst et al., 2010). The balance of the oral microbiota is essential to maintaining human health. Oral dysbiosis is related not only to oral health issues, such as dental caries, periodontal diseases, and tooth loss (Yang et al., 2012; Teles et al., 2013), but also to systemic diseases, such as cardiovascular disease, diabetes, and even cancer (Koren et al., 2011; Galvão-Moreira and da Cruz, 2016; Long et al., 2017). Thus, maintaining normal and healthy oral microbiota is important for human health.

China is the largest producer and consumer of tobacco in the world. Cigarette smoking is a common risk factor affecting public health (Hu et al., 2006). Cigarette smoke contains numerous toxic substances. The oral cavity is the first part of the body that comes into contact with smoke. Thus, the oral microbiota has the greatest potential to be affected by smoke. The toxicants in cigarette smoke can interfere with oral microbial ecology *via* antibiotic effects and oxygen deprivation (Macgregor, 1989).

Cigarette smoking is a cause of oral dysbiosis that affects the diversity of oral microbiota and their functional potential (Mason et al., 2015; Wu et al., 2016; Yu et al., 2017; Yang et al., 2019). In addition, Wu et al. studied the effects of cigarette smoking on oral wash samples among American adults and observed that smoking may affect oral microbiota by promoting an anaerobic oral environment (Wu et al., 2016). Yang et al. investigated cigarette smoking in relation to the oral microbiota of mouth rinse samples in low-income and African-American populations, and they observed that changes in oral microbiota caused by cigarette smoking were recovered after smoking cessation (Yang et al., 2019). Sato et al. observed in East Asians that tongue microbiota and related metagenomic pathways of current smokers differ from those of never smokers (Sato et al., 2020b).

The oral ecological environment is affected by many external factors and exhibits considerable individual differences. For instance, a study determined the distinctiveness of the saliva microbiome of humans living under different climatic conditions (Li et al., 2014). Genetic variations of the host were also reported to influence the oral microbiota (Demmitt et al., 2017). Therefore, the effect of cigarette smoking on the overall oral microbiota may depend on geographic or ethnic background. Epidemiological studies exploring the effect of cigarette smoking on the composition of the oral microbiota in Chinese people remain lacking. Therefore, the relationship between the oral microbiota and cigarette smoking in China warrants further investigation.

The aim of this study was to improve our understanding of the impact of cigarette smoking on the oral microbiota in the Chinese population. In this work, we employed saliva bacterial 16S rRNA gene sequencing to conduct an oral microbial study of 316 subjects from three areas of China. We recruited individuals from three areas in southern, northern and northeastern China with large latitude differences, distinct dietary habits, and different life habits. This study may provide a useful opportunity to further assess the consistent relationship between cigarette smoking and the oral microbiota and add findings from the Chinese population.

## Materials And Methods

### Study Population and Saliva Sample Collection

Three populations from (1) Guangdong Province (defined as the GD population), (2) Yangquan city in Shanxi Province (defined as the YQ population), and (3) Mishan city in Heilongjiang Province (defined as the MS population) were included in our study. These populations have been previously described in detail (He et al., 2019). Briefly, 1223 adults with a mean age (± SD) of 46.89 ± 11.47 years were recruited between 1 October 2015 and 1 August 2016 in the GD population. A total of 2416 adults with a mean age (± SD) of 46.74 ± 11.16 years were recruited between 1 May and 1 October 2014 in the YQ population. A total of 1279 adults with a mean age (± SD) of 46.17 ± 11.48 years were recruited between 1 May and 1 September 2015 in the MS population. At the enrollment step, saliva samples were collected. Informed consent was signed by every subject before the interview, and the Human Ethics Committee of Sun Yat-sen University Cancer Center reviewed and approved the proposal for the study (the approval number: GZR2013-008).

In the present study, we conducted stratified random sampling by age and sex from three populations. A subset of 316 subjects were included in the present study, including 150 from the GD population, 81 from the YQ population, and 85 from the MS population. Comprehensive demographic and lifestyle information was collected using face-to-face interviews conducted by well-trained investigators. Current smokers were defined as subjects who smoked at least one cigarette every one to three days in the past year. Former smokers were defined as subjects who smoked at least one cigarette every one to three days but had quit smoking for at least a year. Never smokers were defined as subjects who had never smoked at least one cigarette every one to three days. We found former and current smokers overlapped on the principal coordinate analysis plot (**Supplementary Figure 1A**). The overall microbiota composition of former smokers tended to be more similar to current smokers than never smokers (**Supplementary Figure 1B**). And given the number of ever smokers in this study is small, in order to increase the statistical power, we combined ever smokers and current smokers into one smokers group. Unstimulated whole saliva samples were collected from participants during study enrollment. All participants were asked not to eat or drink for half an hour before providing samples. Five milliliters of saliva were collected into a 50-ml centrifuge tube. We added an equal volume of salivary lysate to the saliva to facilitate subsequent nucleic acid extraction. The salivary lysate included Tris-HCL (pH=8.0), EDTA, sucrose, NaCl and 10% SDS. Then the saliva samples were divided into 2-ml EP tubes and were subsequently stored at −80°C until use.

### DNA Extraction and 16S rRNA Gene Sequencing

Bacterial genomic DNA was extracted from saliva samples using the Powersoil DNA isolation kit (Qiagen, Duesseldorf, Hilden, Germany) with the bead-beating method according to the manufacturer’s instructions. Amplicon libraries were generated following an optimized protocol based on a previously described method (Gohl et al., 2016) with slight modifications. Briefly, the V4 variable region of the 16S rRNA gene was amplified with forward and reverse primers containing common adapter sequences and 12-bp barcodes: [barcode] + [overhang] + 515F/806R (GTGCCAGCMGCCGCGGTAA/GGACTACHVGGGTWTCTAAT) (Caporaso et al., 2011) with 20 cycles. Next, the Illumina flow cell adapters and dual indices (6 bp) were added in a secondary amplification with 10 cycles of amplification. PCR products were visualized with nucleic acid gel electrophoresis, purified using Agencourt AMPure XP (Beckman Coulter, Brea, CA, USA), and quantified using the Qubit HS kit (Invitrogen, Carlsbad, MA, USA). Pooled amplicon libraries were sequenced using the Illumina MiSeq 250-bp paired-end strategy.

### Sequencing Data Processing and Quality Controls

QIIME2 version 2019.4 was utilized to process and analyze 16S rRNA gene amplicon sequences (Bolyen et al., 2019). Multiplexed libraries were deconvoluted based on the barcodes assigned to each sample. After demultiplexing, quality control and paired‐end read joining were performed with DADA2 (Callahan et al., 2016). Pre-processed sequences were clustered into amplicon sequence variants (ASVs). ASVs observed in fewer than three samples and with total abundances of less than five were excluded. All quality-checked reads were mapped to each OTU with > 99% identity using the Greengenes database 13.8 (McDonald et al., 2012) predefined taxonomy map of reference sequences. To detect possible bacteria in reagents and environmental contamination obtained in the course of the experiment, we used negative control samples in the processes of DNA extraction and construction of the PCR library. After the above steps with QIIME2, read counts from negative control samples were negligible compared to saliva samples (**Supplementary Figure 2**).

### Statistical Analysis

We used chi-square tests for categorical variables and Student’s t tests for continuous variables. A *P*-value < 0.05 was considered to be significant. For the diversity analysis of 16S rRNA data, samples were rarefied to 10000 sequences per sample. The alpha diversity of the saliva microbiota between never smokers and smokers was measured by the Shannon’s diversity index (Lozupone and Knight, 2008). The beta diversity was assessed using weighted UniFrac distance matrices (Lozupone et al., 2007). Permutational multivariate analysis of variance (PERMANOVA; adonis function, vegan package, R) of the weighted UniFrac distance was employed to test differences in overall oral microbiome composition across smoking and nonsmoking groups. Principal coordinate analysis (PCoA) was performed to obtain principal coordinates and visualize complex, multidimensional data. The significance level was *P* < 0.05.

The detection of the difference in the relative abundance of features at the phylum, class, order, family, genus, and species levels between never smokers and smokers was performed using the linear discriminant analysis effect size (LEfSe) method (Segata et al., 2011). We used the online galaxy server (https://huttenhower.sph.harvard.edu/galaxy/) to identify differentially abundant bacterial taxa between never smokers and smokers. Features with logarithmic LDA scores for discriminative features > 2.0 and false discovery rate (FDR)-adjusted *q*-values < 0.05 were considered to be significant. Next, we investigated potential interactions of features at the genus and species levels by network analysis of taxa co-occurrence patterns using SparCC (Friedman and Alm, 2012). Cytoscape (version 3.7.1) was employed to establish the genus-genus and species-species networks. Only Spearman’s correlation coefficients > 0.4 or < −0.4 and with *P*-values < 0.05 are shown.

Phylogenetic Investigation of Communities by Reconstruction of Unobserved States (PICRUSt) (http://galaxy.morganlangille.com/) was used to infer the functional shifts in the microbiota of never smokers and smokers. PICRUSt can predict the Kyoto Encyclopedia of Genes and Genomes (KEGG) pathway functional profiles of microbial communities *via* 16S rRNA gene sequences (Langille et al., 2013). Statistical Analysis of Metagenomic Profiles (STAMP) was employed to compute the abundance differences of KEGG pathways. False discovery rate (FDR)-adjusted *q*-values less than 0.05 were considered to be significant. Next, we used Spearman’s rank correlation to examine the associations between pathways and genera and species that were significantly associated with smoking status. Pathways with average relative abundance > 1% were included.

All statistical tests were two-sided, and all statistical analyses were performed using R version 3.6.3.

## Results

### Characteristics of the Study Participants

Our study included 316 subjects from China who provided valid informed consent, a completed questionnaire on smoking status, and a saliva sample. Our population was recruited from three areas in China, which included 150 from the GD population, 81 from the YQ population, and 85 from the MS population. Demographic characteristics of the subjects in each region were shown in **Supplementary Table 1**. Age, gender, and smoking status were equally comparable among three populations. The education level was significantly different and more people were under high school in GD population.

### Bacterial Diversity and Community Structure of Saliva Microbiota

**Figure 1:**
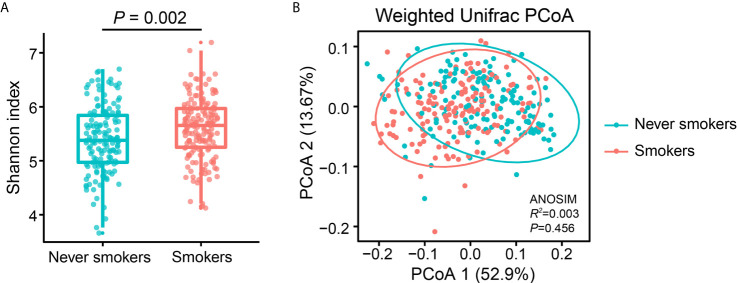
Alpha and beta diversity estimates of the oral microbial community. **(A)** Comparison of Shannon index in the oral microbiota between never smokers and smokers (*P* = 0.002). **(B)** PCoA based on the weighted UniFrac distances of the oral microbial communities between never smokers and smokers.

To investigate the effects of smoking status on oral microbiota diversity, we examined the bacterial diversity of salivary microbiota in different smoking statuses. The Shannon diversity index, an alpha diversity estimator, was significantly higher in smokers than in never smokers (*P* = 0.002) (**Figure 1A**). Next, we performed a principal coordinate analysis based on weighted UniFrac distances to determine whether the overall microbiota composition differed according to smoking status. **Figure 1B** presents a PCoA plot based on the weighted UniFrac distances. Although there was no significant difference between never smokers and smokers in the overall bacterial community structure of saliva according to ANOSIM (*P* = 0.456), we observed separate trends between the two groups.

### Bacterial Taxa With a Significant Differential Abundance Between Never Smokers and Smokers

**Figure 2:**
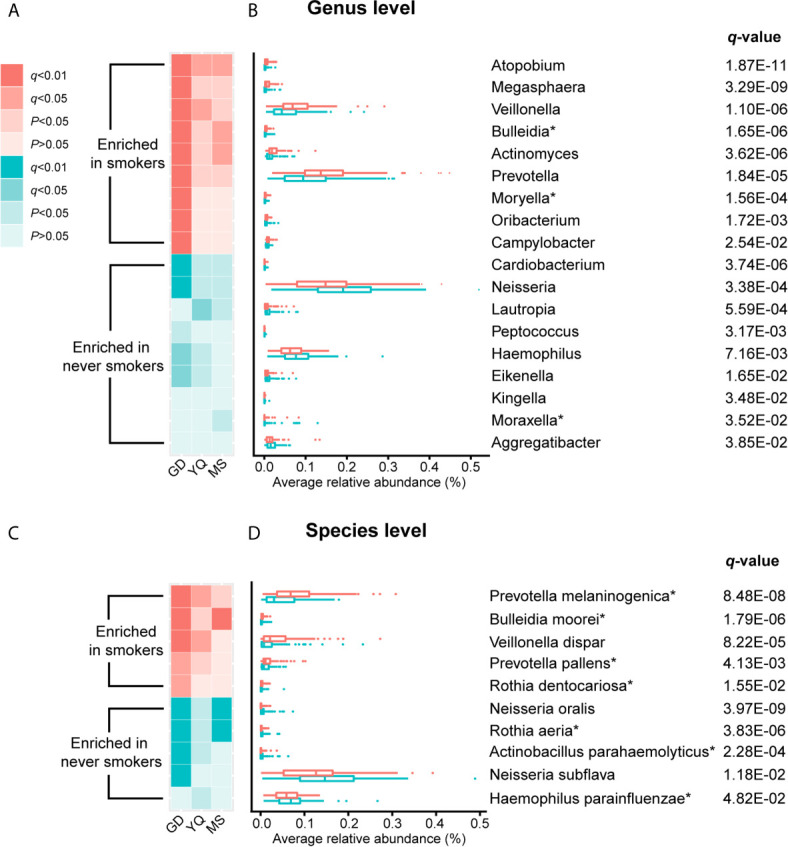
The result of comparison of bacterial abundance at the genus level and species level. **(A, C)** The heatmaps show the *q*-value and *P*-value of these differentially abundant taxa in Guangdong, Yangquan, and Mishan populations. **(B, D)** The box plots show the average relative abundances of differentially abundant taxa in total populations. * Means bacterium has been found firstly to differ significantly between never smokers and smokers in our study.

To further explore the effects of smoking status on specific bacteria, LEfSe analysis was performed to investigate the differentially abundant taxa between never smokers and smokers. We observed 53 differentially abundant taxa between never smokers and smokers that reached significance with a log LDA score > 2.0 and FDR *q*-value < 0.05 in the total population (**Supplementary Table 2**). Among these taxa, there were 18 differentially abundant taxa at the genus level and 10 differentially abundant taxa at the species level (**Table 1** and **Figure 2**).

**Table 1:** Differentially abundant taxa at the genus and species level between never smokers and smokers.

Taxa	Detectable rate (%)		Average relative abundance (%)		LDA score	*q*-value^a^
	Never smokers (n=150)	Smokers (n=166)	Never smokers (n=150)	Smokers (n=166)		
Phylum Actinobacteria						
Genus Actinomyces	100.00	100.00	1.56	2.33	3.60	3.62E-06
Species Rothia aeria	96.67	82.00	0.42	0.21	3.04	3.83E-06
Species Rothia dentocariosa	80.67	88.00	0.26	0.33	2.60	1.55E-02
Genus Atopobium	88.00	97.33	0.23	0.58	3.25	1.87E-11
Phylum Bacteroidetes						
Genus Prevotella	100.00	100.00	10.96	15.06	4.30	1.84E-05
Species Prevotella melaninogenica	100.00	100.00	4.80	8.07	4.19	8.48E-08
Species Prevotella pallens	98.67	100.00	1.23	1.69	3.30	4.13E-03
Phylum Firmicutes						
Genus Moryella	85.33	96.67	0.16	0.25	2.69	1.56E-04
Genus Oribacterium	98.67	100.00	0.44	0.53	2.63	1.72E-03
Genus Peptococcus	83.33	72.67	0.05	0.03	2.10	3.07E-03
Genus Megasphaera	84.00	96.67	0.38	0.82	3.34	3.29E-09
Genus Veillonella	100.00	100.00	5.36	7.79	4.07	1.10E-06
Species Veillonella dispar	90.67	93.33	2.20	3.69	3.86	8.22E-05
Genus Bulleidia	98.67	98.67	0.30	0.44	2.85	1.65E-06
Species Bulleidia moorei	98.67	98.67	0.29	0.42	2.82	1.79E-06
Phylum Proteobacteria						
Genus Lautropia	98.00	96.00	1.06	0.70	3.25	5.59E-04
Genus Eikenella	99.33	100.00	1.06	0.69	3.30	1.65E-02
Genus Kingella	69.33	58.00	0.07	0.04	2.11	3.48E-02
Genus Neisseria	100.00	100.00	19.43	15.25	4.31	3.38E-04
Species Neisseria oralis	93.33	78.67	0.70	0.25	3.36	3.97E-09
Species Neisseria subflava	100.00	100.00	14.96	12.11	4.16	1.18E-02
Genus Campylobacter	100.00	100.00	0.78	0.93	2.87	2.54E-02
Genus Cardiobacterium	88.00	78.00	0.11	0.05	2.43	3.74E-06
Species Actinobacillus parahaemolyticus	64.67	40.00	0.48	0.18	3.17	2.28E-04
Genus Aggregatibacter	98.00	98.00	1.82	1.53	3.18	3.85E-02
Genus Haemophilus	100.00	100.00	8.14	6.53	3.91	7.16E-03
Species Haemophilus parainfluenzae	100.00	100.00	7.19	5.97	3.79	4.82E-02
Genus Moraxella	43.33	20.67	0.54	0.19	3.29	3.52E-02

a False discovery rate adjusted q-values were calcualated based based on P-values from the LefSe analysis.

At the genus level, *Actinomyces, Atopobium, Prevotella, Moryella, Oribacterium, Megasphaera, Veillonella, Bulleidia*, and *Campylobacter* were significantly enriched in smokers. *Peptococcus, Lautropia, Eikenella, Kingella, Neisseria, Cardiobacterium, Aggregatibacter, Haemophilus*, and *Moraxella* were significantly depleted in smokers. Among these genera, *Moryella, Bulleidia*, and *Moraxella* were first observed to be significantly different in smoking status. In addition, at the species level, *Rothia dentocariosa, Prevotella melaninogenica, Prevotella pallens, Veillonella dispar*, and *Bulleidia moorei* were determined to be significantly enriched in smokers. *Rothia aeria, Neisseria oralis, Neisseria subflava, Actinobacillus parahaemolyticus*, and *Haemophilus parainfluenzae* were significantly depleted in smokers. These species, except for *Veillonella dispar, Neisseria oralis*, and *Neisseria subflava*, were first found to have significant differences in smoking status. The bacteria enriched in smokers primarily belonged to the phyla *Actinobacteria, Bacteroidetes*, and *Firmicutes*. Conversely, the bacteria that decreased in smokers primarily belonged to the *Proteobacteria* phylum.

We analyzed the distribution of these bacteria in three populations separately to identify whether the oral taxa consistently altered by cigarette smoking were independent of climate environment and lifestyle (**Supplementary Table 2** and **Figure 2**). For 18 differentially abundant genera and 10 differentially abundant species in the total population, these bacteria showed consistent abundance changes in all three areas. Taking *P* < 0.05 as the standard, 11 genera and 7 species exhibited significant differences in at least two areas. Taking *q* < 0.05 as the standard, 4 genera and 5 species exhibited significant differences in at least two areas. Among these bacteria, *Atopobium* remained significantly different (*q* < 0.05) in the three populations. *Actinomyces*, *Veillonella*, *Bulleidia*, *Rothia aeria*, *Prevotella melaninogenica*, *Veillonella dispar*, *Bulleidia moorei*, and *Neisseria oralis* maintained (*q* < 0.05) significant differences in the two populations. Although the results were not completely consistent across the three different populations, the trend was the same. This finding may be attributable to the small number of people in independent areas, as no significant difference was observed.

### Co-Occurrence Network of the Bacteria in the Saliva Microbiota

**Figure 3:**
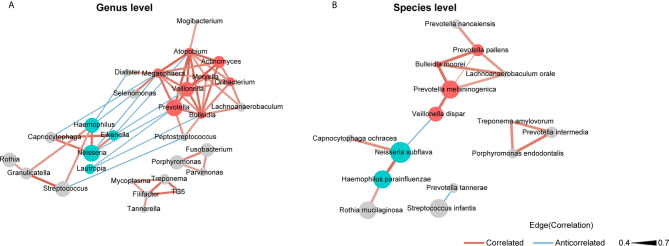
Co-occurrence network of **(A)** the genera and **(B)** the species in oral microbiota. The sizes of the nodes indicate the mean relative abundance of the corresponding bacteria. The red nodes represent bacteria that were enriched in smokers. The blue nodes represent bacteria that were decreased in smokers. The gray nodes represent bacteria that were not identified as being significantly associated to smokers or never smokers. The width of the lines reflects the strength of correlation and the color of the lines, red or blue, indicates a positive or negative correlation, respectively.

We used SparCC and Cytoscape to construct network structures to analyze co-occurrence and co-excluding relationships at the genus and species levels. Genera and species correlations that met the threshold of Spearman’s *r* > 0.4 and *P* < 0.05 are shown in the networks (**Figure 3**). At the genus level (**Figure 3A**), there were 29 nodes and 58 edges (including 48 positive correlations and 10 negative correlations). At the species level (**Figure 3B**), there were 15 nodes and 16 edges (14 positive correlations and 2 negative correlations). Analysis of the bacterial correlation coefficients showed distinct clusters separated by smoking status. From the co-occurrence network, we observed that the bacteria that were significantly enriched in smokers had a strong positive correlation but a negative correlation with the bacteria that were decreased in smokers. At the genus level, *Prevotella*, *Veillonella*, *Atopobium*, *Megasphaera*, and *Bulleidia* were the top five genera with hubs with more than 6 linkers. Notably, all of these genera were smoker-enriched taxa (**Figure 3A**). *Veillonella* showed the strongest correlation with *Actinomyces* (r = 0.70). At the species level, *Prevotella melaninogenica*, *Prevotella pallens*, *Bulleidia moorei*, *Lachnoanaerobaculum orale*, and *Neisseria subflava* were the top five hubs with more than 2 linkers. Among these species, *Prevotella melaninogenica* showed the strongest correlation with *Prevotella pallens* (*r* = 0.72).

### Correlation Between Saliva Microbiota and Predictive Functional Pathways

**Figure 4:**
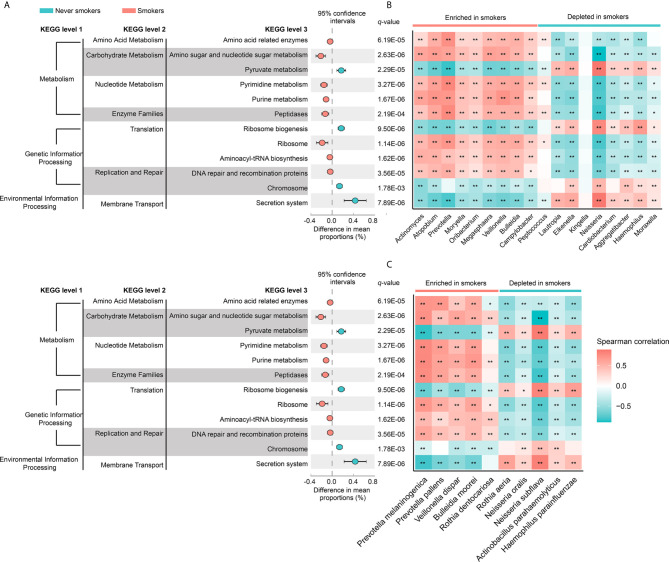
**(A)** The different analysis of microbial community functions between never smokers and smokers (KEGG pathways average relative abundance > 1%). Difference in mean proportion for pathways showing significant difference in abundance are shown. The 95% confidence intervals and statistical significance (FDR *q*-value) are indicated as well. **(B)** Heatmap of spearman correlation between differentially abundant genera and above-mentioned pathways. **(C)** Heatmap of spearman correlation between differentially abundant species and above-mentioned pathways. The strength of the color depicts the Spearman’s correlation coefficients (negative score, blue; positive score, red). *P < 0.05, **P < 0.01.

To investigate the functional role of the oral microbiota in different smoking statuses, PICRUSt analysis was performed to explore microbiome function based on inferred metagenomes. Of 328 KEGG pathways identified, we excluded pathways that occurred in less than 30% of participants and with an average relative abundance below 1%. Twelve discernible microbiota pathways were clearly different between never smokers and smokers (*q* < 0.01, **Supplementary Table 3**). These pathways included pathways related to environmental information processing, genetic information processing, and metabolism. We found that smokers showed a higher abundance of most pathways from genetic information processing and metabolism but showed a lower abundance of pathways associated with environmental information processing (**Figure 4A**). Notably, pathways related to acid production (amino acid-related enzymes and amino sugar and nucleotide sugar metabolism, *q* = 6.19E-05 and *q* = 2.63E-06, respectively) were all enriched in smokers. Next, we analyzed the correlation between differentially abundant pathways and genera/species to explore whether the bacteria altered by cigarette smoking were necessarily related to many of these pathways (**Figures 4B, C** **)**. We observed that genera and species enriched in smokers were positively associated with the KEGG pathways increased in smokers. The bacteria significantly enriched in smokers had similar functions but had a distinct difference in function from those decreased in smokers.

## Discussion

In this study, we compared the saliva microbiota composition of never smokers and smokers, as well as the functional profiles of the saliva samples, using 16S rRNA sequencing data. We observed a clear difference in the saliva microbiota and related metagenomic pathways between never smokers and smokers. Previous studies on the association between cigarette smoking and oral microbiota have primarily focused on non-Asian populations. To the best of our knowledge, this report describes the first relatively systematic study to demonstrate cigarette smoking-associated oral microbial alterations in a Chinese population.

In the present study, the influence of cigarette smoking on the diversity and overall composition of oral microbiota was analyzed. We found that the Shannon diversity index was different between never smokers and smokers, and the difference was significant. This result indicated differences in richness and evenness between never smokers and smokers. Beta diversity exhibited separate trends but no significant difference between the two groups. Our findings suggested that the oral microbiota of former smokers was more similar to that of current smokers than never smokers. This suggested that the effects of cigarette smoking on oral microbiota may persist for years. In addition, we found that the majority of the observed taxa were present in both the never smoker and smoker groups, albeit at different frequencies of detection. Fifty-three taxa were found to be significantly different between never smokers and smokers, including 18 genera and 10 species. These taxa were primarily distributed in four bacterial phyla: *Actinobacteria*, *Bacteroidetes*, *Firmicutes*, and *Proteobacteria*. This observation was in accordance with previous studies (Wu et al., 2016; Yang et al., 2019). At the genus level, most of the differentially abundant taxa that we identified were consistent with those observed in previous studies (Camelo-Castillo et al., 2015; Wu et al., 2016; Yang et al., 2019; Sato et al., 2020b; Wirth et al., 2020), except for *Moryella, Bulleidia*, and *Moraxella*. The previous 16S rRNA gene sequences on cigarette smoking and oral microbiota have been less thoroughly investigated at the species level. *Rothia aeria*, *Rothia dentocariosa*, *Prevotella melaninogenica*, *Prevotella pallens*, *Bulleidia moorei*, *Actinobacillus parahaemolyticus*, and *Haemophilus parainfluenzae* were all first identified in our study, and *Veillonella dispar* (Sato et al., 2020a), *Neisseria oralis*, and *Neisseria subflava* (Yang et al., 2019) were not.

To further explore whether the influence of cigarette smoking on saliva microbiota was stable in different populations from distinct environments, we performed LEfSe analysis in GD, YQ, and MS populations separately. We found a consistent alteration trend of the abundance of taxa according to smoking status, although some of them could not reach a significant *P*-value. We still found that 11 genera and 7 species had significant differences in at least two areas at the standard of *P* < 0.05, indicating that the effect of cigarette smoking on oral microbiota was stable even under different climatic conditions and living habits. *Atopobium* remained significantly different in all three populations with *q* < 0.05, indicating that *Atopobium* may be the bacterium that is most affected by cigarette smoking.

The relationships between diseases and oral microbiota were previously investigated in several studies. We found that the bacteria associated with diseases were primarily increased in smokers. For example, *Actinomyces* contributed to the development of oral diseases, such as caries and periodontitis (Kolenbrander, 2000). *Actinomyces* in saliva microbiota was also reported to be related to liver cancer progression. (Li et al., 2020). A previous study showed the association between *Atopobium* in tissue and oral squamous cell carcinoma (Perera et al., 2018). Another study showed that *Actinomyces* and *Atopobium* in saliva were both related to a high risk of esophageal squamous cell carcinoma (Wang et al., 2019). In pancreatic head carcinoma (Lu et al., 2019), *Actinomyces* and *Atopobium* were overrepresented in the tongue coating. By studying the saliva microbiota of inflammatory bowel disease patients, *Atopobium* was significantly increased (Qi et al., 2020). The abundances of *Prevotella*, *Veillonella*, *Megasphaera*, *Atopobium*, and *Oribacterium* were all increased in saliva samples from reflux esophagitis patients (Wang et al., 2020). In addition, at the species level, *Prevotella melaninogenica* and *Prevotella pallens* were associated with oral squamous cell carcinoma (Pushalkar et al., 2012). *Veillonella dispar* was significantly increased in the intestinal flora of sporadic nasopharyngeal carcinoma patients (Jiang et al., 2019). Cigarette smoking may affect the health of smokers by affecting these bacteria.

Notably, bacteria that significantly increased in smokers were anaerobes, and those that decreased were aerobes. This phenomenon might be related to the formation of an oral oxygen deprivation state caused by cigarette smoking (Macgregor, 1989). Cigarette smoking may create an oxygen-free environment in the mouth. This effect would influence the oxygen availability of microbes in the oral cavity, ultimately altering the oral microbial ecology. Oral bacteria can convert nitrate, which is abundant in vegetables, to nitrite, which may make the oral cavity more acidic (Li et al., 2007). Anaerobic bacteria promote this conversion, especially *Actinomyces* and *Veillonella* (Hyde et al., 2014). Notably, Actinomyces and Veillonella were found to be significantly enriched in smokers and had the strongest correlation at the genus level in our study. The acid environment promotes the development of biofilms in the oral cavity and is related to disease of the oral cavity (Krzyściak et al., 2013).

The oral microbiota also plays a key role in the metabolism and degradation of amino acids and carbohydrates. Analysis of inferred metagenomes indicated that pathways with significant differences between never smokers and smokers mainly belong to the metabolism category. Additionally, we found that bacteria that were significantly enriched in smokers had opposite functions to those bacteria depleted in smokers. This finding suggested that cigarette smoking may affect oral health by altering the microbiota and their metabolic functions. We found positive correlations between bacteria enriched in smokers and amino acid-related enzymes and pathways of amino sugar and nucleotide sugar metabolism pathways, which would increase the acidity of the oral cavity environment. Consistent with our findings, Li et al. also found that ribosome, DNA repair and recombination proteins, and purine metabolism were increased in the mouse lower respiratory tract microbiome when exposed to cigarette smoke (Li et al., 2019). This finding suggests that cigarette smoking could affect the microbiota not only in the oral cavity but also in the lower respiratory tract along the respiratory tract.

Co-occurrence networks can provide insights into the potential interactions in oral microbiota communities. In this study, we found that the bacteria enriched in smokers promoted each other and directly or indirectly suppressed the bacteria depleted in smokers. This finding is probably observed because cigarette smoking creates different oral cavity environments that are better for the bacteria that are enriched in smokers. It is also possible that the bacteria enriched in smokers by other biological mechanisms inhibit the proliferation of bacteria depleted in smokers. Cigarette smoking may affect the oral microbiota by affecting the complex relationships between bacteria.

To the best of our knowledge, our study is the first relatively systematic report to demonstrate the effects of cigarette smoking on the oral microbiota composition in a Chinese population. Our methods relied on high-throughput next-generation sequencing of the 16S rRNA marker gene determined in unstimulated saliva samples. The results of our study are largely consistent with previous studies, although different studies used different oral samples, including oral wash samples, subgingival plaques, and tongue-coating samples, (Camelo-Castillo et al., 2015; Wu et al., 2016; Sato et al., 2020b). The microbiota from different oral cavity sites were reported to be highly similar, although differences exist with a small effect size (Hall et al., 2017). Another study also found no differences in the effects of cigarette smoking on the oral cavity and nasal cavity microbiota compositions (Yu et al., 2017). In fecal samples, we found that the effects of cigarette smoking on the gut microbiota were similar to those on the oral microbiota (Capurso and Lahner, 2017; Nolan-Kenney et al., 2019). Whether the harmful substances in cigarettes directly affect the microbiota of different body parts or whether the bacteria in the oral cavity migrate to other locations along with the respiratory tract or digestive tract needs further research.

A limitation of our study is the small sample size, limiting our ability to detect potential differences in overall oral microbiome composition. Due to the small sample size of former smokers, we combined them with current smokers into one group of smokers to increase statistical power. This made us unable to further discover the effects of smoking cessation on oral microbiota. Future studies should investigate the effects of cigarette smoking on oral microbiota in a larger sample size. Additionally, although our findings suggested that cigarette smoking may make the oral environment more acidic, it is unable to objectively measure salivary pH to test our hypothesis due to the addition of lysates that can affect the pH of saliva. Further studies are needed to confirm this hypothesis through a better research design.

In summary, in this study of the oral microbiota in a Chinese population, we observed that cigarette smoking influenced the overall oral microbiota community composition and the abundance of specific oral taxa. Our study suggested that cigarette smoking may affect health by creating a different environment in the oral cavity by affecting complex relationships between bacteria and by altering certain metabolic pathways. Future studies are still warranted to investigate the impact of cigarette smoking on the metagenomic content of the microbiome in multiple parts of the body under a relatively larger sample size to enhance our understanding of the systematic microbiota-related effects of cigarette smoking, which might provide new evidence for microbiota-targeted approaches for disease prevention.

## DATA AVAILABILITY STATEMENT

The data presented in the study are deposited in the NCBI BioProject repository, accession numbers: PRJNA720269 and PRJNA721325.

## ETHICS STATEMENT

The studies involving human participants were reviewed and approved by the Human Ethics Committee of Sun Yat-sen University Cancer Center (the approval number: GZR2013-008). An informed consent was signed by every subject before the interview.

## AUTHOR CONTRIBUTIONS

W-HJ, YL, and Y-JJ conceived and designed the study. W-HJ, J-BZ, W-QX, and Y-QH supported the administrative work of the study. W-HJ, W-QX, Y-QH, M-QZ, X-TT, L-LY, and W-LZ contributed to population enrollment and data collection. YL and Y-JJ contributed to data analysis. All authors contributed to the article and approved the submitted version.

## FUNDING

This work was supported by the National Key Research and Development Program of China (grant number 2016YFC1302700), the National Natural Science Foundation of China (grant number 81973131), the Science and Technology Planning Project of Guangzhou, China (grant number 201904010467), the Science and Technology Planning Project of Guangdong Province, China (grant number 2019B030316031), and the Sino-Sweden Joint Research Program (grant number 81861138006).

## Conflict of Interest

The authors declare that the research was conducted in the absence of any commercial or financial relationships that could be construed as a potential conflict of interest.
