# Supplementary material for: Association Between Oral Microbiota and Cigarette Smoking in the Chinese Population
Source: Front Cell Infect Microbiol. 2021 May 28;11:658203. doi: 10.3389/fcimb.2021.658203 (PMC8195269; doi:10.3389/fcimb.2021.658203)
Supplement: Supplementary file 1 [file DataSheet_1.docx]

***Supplementary Material***

**
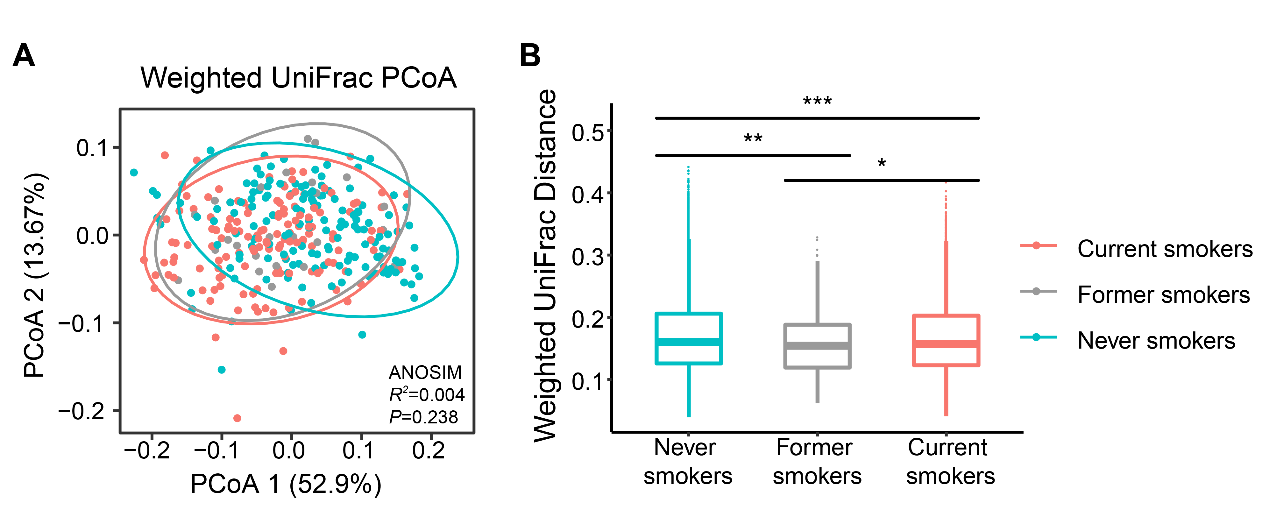
**

**Supplementary Figure 1.** (**A**) Oral microbiome composition according to smoking status (current, former and never) in 316 subjects. The principal coordinate analysis was conducted based on the weighted UniFrac distance. (**B**) Comparison of within group distances for all smoking status indicated that current and former smokers are more alike than are the never smokers. **P* < 0.05, ***P* < 0.01,****P* < 0.001.


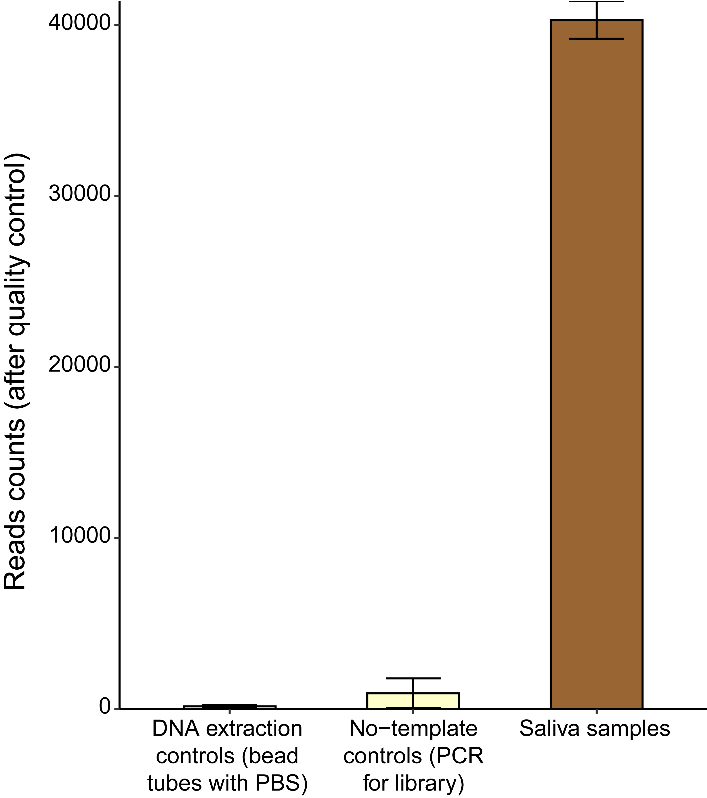


**Supplementary Figure 2.** Comparison of reads counts between negative control samples and saliva samples. There are nine DNA extraction controls and six no-template controls.

| \| \| **Supplementary Table 1**. Demographic characteristics of the subjects in each region \| \| \| \| \| \| \|  \| \| --- \| --- \| --- \| --- \| --- \| --- \| --- \| --- \| \|  \| **Guangdong** \| \| **Yangquan** \| \| **Mishan** \| \| ***P* value** \| \| N=150 \| % \| N=81 \| % \| N=85 \| % \| \| **Age(years)**^a^ \| 150 \| 46.93±10.33 \| 81 \| 47.35±11.28 \| 85 \| 46.01±10.02 \| 0.88^b^ \| \| **Gender** \|  \|  \|  \|  \|  \|  \|  \| \| Male \| 106 \| 70.67 \| 58 \| 71.60 \| 60 \| 70.59 \| 0.99^c^ \| \| Female \| 44 \| 29.33 \| 23 \| 28.40 \| 25 \| 29.41 \|  \| \| **Education** \|  \|  \|  \|  \|  \|  \|  \| \| <High school \| 118 \| 78.67 \| 38 \| 46.91 \| 30 \| 35.29 \| 2.84E-11^c^ \| \| ≥High school \| 32 \| 21.33 \| 43 \| 53.09 \| 55 \| 64.71 \|  \| \| **Smoking status** \|  \|  \|  \|  \|  \|  \|  \| \| Never \| 63 \| 42.00 \| 42 \| 51.85 \| 45 \| 52.94 \| 0.45^c^ \| \| Former \| 13 \| 8.67 \| 7 \| 8.64 \| 7 \| 8.24 \|  \| \| Current \| 74 \| 49.33 \| 32 \| 39.51 \| 33 \| 38.82 \|  \|   ^a^For age, mean±SD were presented.  ^b^*P*-values were based on ANOVA test.  ^c^*P*-values were based on Pearson's Chi-squared test. \| \| --- \| --- \| --- \| --- \| --- \| --- \| --- \| --- \| --- \| --- \| --- \| --- \| --- \| --- \| --- \| --- \| --- \| --- \| --- \| --- \| --- \| --- \| --- \| --- \| --- \| --- \| --- \| --- \| --- \| --- \| --- \| --- \| --- \| --- \| --- \| --- \| --- \| --- \| --- \| --- \| --- \| --- \| --- \| --- \| --- \| --- \| --- \| --- \| --- \| --- \| --- \| --- \| --- \| --- \| --- \| --- \| --- \| --- \| --- \| --- \| --- \| --- \| --- \| --- \| --- \| --- \| --- \| --- \| --- \| --- \| --- \| --- \| --- \| --- \| --- \| --- \| --- \| --- \| --- \| --- \| --- \| --- \| --- \| --- \| --- \| --- \| --- \| --- \| --- \| --- \| --- \| --- \| --- \| --- \| --- \| --- \| --- \| --- \| --- \| --- \| --- \| --- \| --- \| --- \| --- \| --- \| --- \| --- \| --- \| --- \| --- \| |
| --- | --- | --- | --- | --- | --- | --- | --- | --- | --- | --- | --- | --- | --- | --- | --- | --- | --- | --- | --- | --- | --- | --- | --- | --- | --- | --- | --- | --- | --- | --- | --- | --- | --- | --- | --- | --- | --- | --- | --- | --- | --- | --- | --- | --- | --- | --- | --- | --- | --- | --- | --- | --- | --- | --- | --- | --- | --- | --- | --- | --- | --- | --- | --- | --- | --- | --- | --- | --- | --- | --- | --- | --- | --- | --- | --- | --- | --- | --- | --- | --- | --- | --- | --- | --- | --- | --- | --- | --- | --- | --- | --- | --- | --- | --- | --- | --- | --- | --- | --- | --- | --- | --- | --- | --- | --- | --- | --- | --- | --- | --- | --- |

| **Supplementary Table 2.** Differentially abundant taxa between never smokers and smokers | | | | | | | | | | | | | |
| --- | --- | --- | --- | --- | --- | --- | --- | --- | --- | --- | --- | --- | --- |
| **Taxa** | **Total** | | | **Guangdong** | | | **Yangquan** | | | | **Mishan** | | |
|  | Average relative abundance  (%) | | *q*-value^a^ | Average relative abundance  (%) | | *q*-value^a^ | Average relative abundance  (%) | | | *q*-value^a^ | Average relative abundance  (%) | | *q*-value^a^ |
|  | Never smokers  (n=150) | Smokers  (n=166) |  | Never smokers  (n=63) | Smokers  (n=87) |  | Never smokers  (n=42) | | Smokers  (n=39) |  | Never smokers  (n=45) | Smokers  (n=40) |  |
| Phylum Actinobacteria |  |  |  |  |  |  |  |  | |  |  |  |  |
| Family Actinomycetaceae | 1.58 | 2.35 | 3.74E-06 | 1.15 | 1.91 | 1.06E-04 | 1.42 | 1.81 | | 1.21E-01 | 2.39 | 3.82 | 2.29E-02 |
| Genus Actinomyces | 1.56 | 2.33 | 3.62E-06 | 1.13 | 1.90 | 1.07E-04 | 1.39 | 1.77 | | 1.22E-01 | 2.35 | 3.79 | 2.29E-02 |
| Species Rothia aeria | 0.42 | 0.21 | 3.83E-06 | 0.47 | 0.25 | 1.48E-03 | 0.31 | 0.18 | | 1.33E-01 | 0.47 | 0.15 | 9.73E-03 |
| Species Rothia dentocariosa | 0.26 | 0.33 | 1.55E-02 | 0.20 | 0.38 | 1.02E-02 | 0.35 | 0.32 | | 1.76E-01 | 0.27 | 0.23 | 7.21E-01 |
| Family Coriobacteriaceae | 0.26 | 0.63 | 2.11E-11 | 0.16 | 0.54 | 1.74E-07 | 0.24 | 0.56 | | 2.51E-02 | 0.42 | 0.92 | 1.72E-02 |
| Genus Atopobium | 0.23 | 0.58 | 1.87E-11 | 0.13 | 0.49 | 1.04E-07 | 0.19 | 0.47 | | 2.85E-02 | 0.39 | 0.89 | 1.37E-02 |
| Phylum Bacteroidetes | 22.22 | 26.79 | 1.07E-04 | 19.73 | 24.42 | 3.75E-03 | 25.18 | 32.27 | | 5.13E-02 | 22.70 | 26.55 | 2.88E-01 |
| Order Bacteroidales | 20.44 | 25.41 | 6.57E-05 | 17.37 | 22.66 | 1.32E-03 | 23.47 | 31.30 | | 4.16E-02 | 21.71 | 25.55 | 2.81E-01 |
| Genus Prevotella | 10.96 | 15.06 | 1.84E-05 | 8.58 | 13.05 | 6.03E-04 | 12.79 | 18.93 | | 5.69E-02 | 12.53 | 15.46 | 2.86E-01 |
| Species Prevotella melaninogenica | 4.80 | 8.07 | 8.48E-08 | 3.60 | 7.34 | 9.07E-06 | 5.27 | 9.23 | | 3.36E-02 | 6.05 | 8.48 | 2.12E-01 |
| Species Prevotella pallens | 1.23 | 1.69 | 4.13E-03 | 0.95 | 1.45 | 1.73E-02 | 1.09 | 1.86 | | 1.91E-01 | 1.74 | 2.02 | 2.05E-01 |
| Order Flavobacteriales | 1.77 | 1.38 | 2.62E-02 | 2.36 | 1.76 | 4.13E-02 | 1.70 | 0.97 | | 5.37E-02 | 0.99 | 1.00 | 5.44E-01 |
| Family Weeksellaceae | 0.41 | 0.21 | 2.27E-06 | 0.41 | 0.22 | 1.43E-04 | 0.57 | 0.24 | | 6.49E-02 | 0.28 | 0.17 | 5.27E-02 |
| Phylum Firmicutes | 23.60 | 26.01 | 1.39E-02 | 21.76 | 25.08 | 1.86E-02 | 23.60 | 25.62 | | 1.68E-01 | 26.52 | 28.73 | 1.90E-01 |
| Family Gemellaceae | 0.62 | 0.46 | 4.27E-02 | 0.68 | 0.51 | 6.02E-02 | 0.55 | 0.33 | | 1.24E-01 | 0.60 | 0.51 | 6.53E-01 |
| Order Clostridiales | 10.29 | 13.58 | 3.87E-06 | 8.14 | 11.95 | 3.52E-05 | 10.08 | 14.45 | | 4.74E-02 | 13.68 | 16.47 | 2.90E-01 |
| Family Lachnospiraceae | 1.38 | 1.56 | 2.51E-02 | 1.21 | 1.55 | 1.50E-02 | 1.47 | 1.36 | | 7.12E-01 | 1.58 | 1.78 | 1.11E-01 |
| Genus Moryella | 0.16 | 0.25 | 1.56E-04 | 0.14 | 0.28 | 4.91E-05 | 0.14 | 0.13 | | 9.66E-01 | 0.21 | 0.32 | 7.39E-02 |
| Genus Oribacterium | 0.44 | 0.53 | 1.72E-03 | 0.38 | 0.54 | 2.92E-03 | 0.37 | 0.38 | | 3.26E-01 | 0.60 | 0.65 | 8.44E-02 |
| Genus Peptococcus | 0.05 | 0.03 | 3.07E-03 | 0.04 | 0.02 | 7.81E-02 | 0.08 | 0.05 | | 1.97E-01 | 0.05 | 0.04 | 5.38E-02 |
| Family Veillonellaceae | 6.70 | 9.65 | 7.57E-07 | 5.00 | 8.04 | 4.37E-05 | 6.20 | 10.87 | | 1.16E-02 | 9.66 | 12.10 | 2.80E-01 |
| Genus Megasphaera | 0.38 | 0.82 | 3.29E-09 | 0.20 | 0.77 | 1.10E-07 | 0.39 | 0.86 | | 8.40E-02 | 0.63 | 0.93 | 1.25E-01 |
| Genus Veillonella | 5.36 | 7.79 | 1.10E-06 | 4.10 | 6.39 | 1.52E-04 | 4.54 | 8.57 | | 2.36E-02 | 7.94 | 10.15 | 2.91E-01 |
| Species Veillonella dispar | 2.20 | 3.69 | 8.22E-05 | 1.08 | 2.65 | 3.26E-03 | 1.93 | 5.08 | | 1.33E-02 | 4.05 | 4.69 | 1.56E-01 |
| Family Erysipelotrichaceae | 0.31 | 0.46 | 2.01E-06 | 0.23 | 0.37 | 1.98E-04 | 0.32 | 0.38 | | 1.24E-01 | 0.43 | 0.75 | 6.64E-03 |
| Genus Bulleidia | 0.30 | 0.44 | 1.65E-06 | 0.23 | 0.36 | 2.37E-04 | 0.30 | 0.35 | | 1.22E-01 | 0.41 | 0.74 | 1.01E-02 |
| Species Bulleidia moorei | 0.29 | 0.42 | 1.79E-06 | 0.22 | 0.34 | 2.58E-04 | 0.29 | 0.33 | | 1.23E-01 | 0.40 | 0.71 | 8.27E-03 |
| Phylum Proteobacteria | 35.33 | 26.96 | 5.62E-07 | 39.43 | 30.15 | 4.86E-05 | 34.35 | 25.34 | | 5.08E-02 | 30.33 | 21.28 | 2.93E-02 |
| Class Betaproteobacteria | 22.09 | 16.88 | 3.76E-05 | 26.69 | 20.06 | 2.32E-04 | 20.05 | 14.60 | | 1.03E-01 | 17.40 | 11.77 | 4.82E-02 |
| Order Burkholderiales | 1.25 | 0.77 | 1.65E-05 | 1.18 | 0.92 | 9.15E-02 | 1.59 | 0.71 | | 1.43E-02 | 1.04 | 0.51 | 1.82E-02 |
| Family Burkholderiaceae | 1.07 | 0.70 | 4.18E-04 | 1.04 | 0.85 | 1.46E-01 | 1.40 | 0.62 | | 2.46E-02 | 0.84 | 0.48 | 5.81E-02 |
| Genus Lautropia | 1.06 | 0.70 | 5.59E-04 | 1.04 | 0.85 | 1.46E-01 | 1.37 | 0.62 | | 3.06E-02 | 0.83 | 0.48 | 5.90E-02 |
| Family Comamonadaceae | 0.18 | 0.07 | 4.26E-04 | 0.14 | 0.07 | 1.73E-02 | 0.18 | 0.09 | | 1.63E-01 | 0.20 | 0.03 | 1.58E-01 |
| Family Neisseriaceae | 20.84 | 16.10 | 1.23E-04 | 25.51 | 19.14 | 2.55E-04 | 18.46 | 13.88 | | 1.14E-01 | 16.36 | 11.25 | 7.41E-02 |
| Genus Eikenella | 1.06 | 0.69 | 1.65E-02 | 1.12 | 0.73 | 4.15E-02 | 1.42 | 0.87 | | 5.62E-02 | 0.56 | 0.38 | 2.31E-01 |
| Genus Kingella | 0.07 | 0.04 | 3.48E-02 | 0.07 | 0.03 | 1.31E-01 | 0.07 | <0.01 | | 2.10E-01 | 0.05 | 0.05 | 5.93E-01 |
| Genus Neisseria | 19.43 | 15.25 | 3.38E-04 | 24.04 | 18.24 | 6.58E-04 | 16.58 | 12.84 | | 1.71E-01 | 15.58 | 10.80 | 7.33E-02 |
| Species Neisseria oralis | 0.70 | 0.25 | 3.97E-09 | 0.54 | 0.15 | 5.99E-05 | 0.87 | 0.35 | | 9.09E-02 | 0.55 | 0.12 | 8.92E-03 |
| Species Neisseria subflava | 14.96 | 12.11 | 1.18E-02 | 18.93 | 14.55 | 3.98E-03 | 12.44 | 10.26 | | 1.30E-01 | 11.95 | 8.56 | 1.99E-01 |
| Order Campylobacterales | 0.79 | 0.93 | 2.74E-02 | 0.69 | 0.89 | 1.05E-02 | 0.89 | 0.93 | | 9.40E-01 | 0.79 | 1.00 | 2.75E-01 |
| Genus Campylobacter | 0.78 | 0.93 | 2.54E-02 | 0.68 | 0.89 | 8.54E-03 | 0.88 | 0.91 | | 9.17E-01 | 0.79 | 0.99 | 2.74E-01 |
| Class Gammaproteobacteria | 12.20 | 9.07 | 5.00E-06 | 11.99 | 9.12 | 2.55E-04 | 13.28 | 9.71 | | 1.18E-01 | 11.51 | 8.46 | 1.55E-01 |
| Family Cardiobacteriaceae | 0.12 | 0.05 | 3.23E-06 | 0.11 | 0.05 | 1.53E-03 | 0.16 | 0.07 | | 1.22E-01 | 0.09 | 0.05 | 4.79E-02 |
| Genus Cardiobacterium | 0.11 | 0.05 | 3.74E-06 | 0.09 | 0.05 | 2.15E-03 | 0.16 | 0.06 | | 1.17E-01 | 0.09 | 0.05 | 5.02E-02 |
| Family Pasteurellaceae | 11.04 | 8.51 | 8.18E-05 | 10.54 | 8.31 | 7.33E-04 | 12.92 | 9.38 | | 1.17E-01 | 9.98 | 8.27 | 1.64E-01 |
| Species Actinobacillus parahaemolyticus | 0.48 | 0.18 | 2.28E-04 | 0.41 | 0.12 | 1.87E-03 | 0.73 | 0.30 | | 7.01E-02 | 0.21 | 0.16 | 5.70E-01 |
| Genus Aggregatibacter | 1.82 | 1.53 | 3.85E-02 | 1.78 | 1.42 | 2.23E-01 | 2.12 | 2.28 | | 2.45E-01 | 1.60 | 1.04 | 2.74E-01 |
| Genus Haemophilus | 8.14 | 6.53 | 7.16E-03 | 7.91 | 6.57 | 4.13E-02 | 9.21 | 6.31 | | 8.60E-02 | 7.57 | 6.80 | 5.44E-01 |
| Species Haemophilus parainfluenzae | 7.19 | 5.97 | 4.82E-02 | 7.18 | 6.31 | 1.52E-01 | 7.48 | 4.91 | | 7.55E-02 | 7.05 | 6.38 | 6.41E-01 |
| Order Pseudomonadales | 0.67 | 0.21 | 3.00E-03 | 0.84 | 0.24 | 7.12E-02 | 0.17 | 0.24 | | 2.04E-01 | 0.92 | 0.12 | 2.90E-01 |
| Family Moraxellaceae | 0.67 | 0.21 | 1.75E-03 | 0.84 | 0.24 | 7.22E-02 | 0.16 | 0.24 | | 8.82E-02 | 0.92 | 0.11 | 5.31E-02 |
| Genus Moraxella | 0.54 | 0.19 | 3.52E-02 | 0.83 | 0.23 | 1.24E-01 | 0.10 | 0.20 | | 5.89E-01 | 0.53 | 0.08 | 6.66E-02 |
| Phylum TM7 |  |  |  |  |  |  |  |  | |  |  |  |  |
| Class TM7-3 | 1.68 | 2.12 | 2.26E-04 | 1.73 | 2.19 | 2.97E-02 | 1.56 | 2.01 | | 1.73E-01 | 1.69 | 2.05 | 5.06E-02 |
| ^a^False discovery rate adjusted *q*-values were calculated based on *P*-values from the LefSe analysis. | | | | | | | | | | | | | |

| **Supplementary Table 3** Differentially abundant KEGG pathways between never smokers and smokers | | | |
| --- | --- | --- | --- |
| **KEGG pathway** | Average relative abundance  (%) | | *q-*value^a^ |
|  | Never smokers(n=150) | Smokers  (n=166) |  |
| Environmental Information Processing; Membrane Transport; Secretion system | 1.52 | 1.43 | 7.89E-06 |
| Genetic Information Processing; Replication and Repair; Chromosome | 1.77 | 1.76 | 1.78E-03 |
| Genetic Information Processing; Replication and Repair; DNA repair and recombination proteins | 3.28 | 3.32 | 3.56E-05 |
| Genetic Information Processing; Translation; Aminoacyl-tRNA biosynthesis | 1.41 | 1.44 | 1.62E-06 |
| Genetic Information Processing; Translation; Ribosome | 2.86 | 2.92 | 1.14E-06 |
| Genetic Information Processing; Translation; Ribosome biogenesis | 1.75 | 1.73 | 9.50E-06 |
| Metabolism; Amino Acid Metabolism; Amino acid related enzymes | 1.64 | 1.67 | 6.19E-05 |
| Metabolism; Carbohydrate Metabolism; Amino sugar and nucleotide sugar metabolism | 1.20 | 1.26 | 2.63E-06 |
| Metabolism; Carbohydrate Metabolism; Pyruvate metabolism | 1.07 | 1.04 | 2.29E-05 |
| Metabolism; Enzyme Families; Peptidases | 1.85 | 1.90 | 2.19E-04 |
| Metabolism; Nucleotide Metabolism; Purine metabolism | 2.59 | 2.64 | 1.67E-06 |
| Metabolism; Nucleotide Metabolism; Pyrimidine metabolism | 2.03 | 2.08 | 3.27E-06 |
| ^a^False discovery rate adjusted *q*-values were calculated based on *P*-values from from the LefSe analysis. | | | |
